# Silk Fibroin Microparticle/Carboxymethyl Cellulose Composite Gel for Wound Healing Applications

**DOI:** 10.3390/biomimetics10070434

**Published:** 2025-07-02

**Authors:** Alexander Pashutin, Ekaterina Podbolotova, Luidmila Kirsanova, Onur Dosi, Anton E. Efimov, Olga Agapova, Igor Agapov

**Affiliations:** 1 Academician V.I. Shumakov National Medical Research Center of Transplantology and Artificial Organs, Ministry of Health of the Russian Federation, 123182 Moscow, Russia; pashutin.ar@phystech.edu (A.P.); dosi.o@phystech.edu (O.D.);; 2 Moscow Institute of Physics and Technology, 141700 Dolgoprudny, Russia

**Keywords:** silk fibroin microparticles, carboxymethyl cellulose, composite hydrogel, wound healing, cytocompatibility

## Abstract

Silk fibroin has recently gained considerable attention as a promising biomaterial for use in medical and bioengineering technologies due to its biocompatibility and favorable mechanical properties. In this study, composite gel based on silk fibroin microparticles and carboxymethyl cellulose was developed, characterized by a viscous, homogeneous white mass containing uniformly distributed fibroin microparticles ranging from 1 to 20 μm in size. The gel exhibited a kinematic viscosity of 36.5 × 10^−6^ St, allowing for convenient application to wounds using a syringe or spatula while preventing uncontrolled spreading. The cytocompatibility of the gel was confirmed using the methylthiazol tetrazolium (MTT) assay, which showed no cytotoxic effects on 3T3 fibroblast cells. Furthermore, the gel remained stable for over one year when stored at 10 °C, in contrast to conventional fibroin solutions, which typically lose stability within a month under similar conditions. In a full-thickness skin wound model in rats, the application of the gel significantly accelerated skin regeneration, with complete wound closure observed by day 15, compared with 30 days in the control group. Histological analysis confirmed the restoration of all skin layers. These findings demonstrate the high potential of the gel for applications in regenerative medicine and tissue engineering.

## 1. Introduction

The development of novel biomaterials for tissue engineering and regenerative medicine is one of the key challenges in modern biochemistry and biotechnology. In this context, silk has emerged as a promising material due to its low toxicity, availability, ability to be processed into various forms, and tunable properties achieved through chemical modification [1,2,3,4,5]. Silk fiber is primarily composed of two proteins: the structural protein fibroin and the adhesive protein sericin. In raw silk, sericin envelops and binds two adjacent fibroin molecules. Although both components exhibit low toxicity [6], they must be separated to obtain alternative material forms, such as gels, solutions, films, or porous scaffolds, due to their differing solubilities and plasticization requirements: sericin is hydrophilic and readily dissolves in mildly alkaline solutions [7], whereas fibroin contains hydrophobic regions and requires other specific dissolution methods [8,9,10,11,12]. Fibroin, comprising approximately 75% of the total silk mass, is primarily responsible for the material’s remarkable mechanical strength and chemical stability, while also offering the advantage of biodegradability [13,14,15,16,17].

Silk fibroin is a fibrous, semi-crystalline protein whose structure provides it with exceptional mechanical strength. It consists of heavy (H) and light (L) chains covalently linked by a disulfide bond at the C-terminus of the H-chain. The H-chain contains hydrophobic domains, including repetitive sequences such as Gly-Ala-Gly-Ala-Gly-Ser and Gly-Ala/Ser/Tyr, which are capable of forming stable antiparallel β-sheet crystallites. In contrast, the L-chain lacks repetitive sequences, making it more hydrophilic and flexible. The amphiphilic nature of fibroin, with both hydrophobic and hydrophilic regions, enables the formation of micelles in aqueous solutions under specific conditions [3,18,19,20,21].

Silk fibroin can adopt three primary crystalline polymorphs: metastable Silk I (random coil or helical conformation), thermodynamically stable Silk II (antiparallel β-sheet structure stabilized by hydrogen bonding), and interfacial Silk III, which forms at the water–air boundary and exhibits limited stability [3,14,15].

These unique physicochemical properties make fibroin suitable for medical applications in various materials and solution-based forms [6,11,12,22,23,24]. It has been broadly applied in various biomedical formats, such as bioactive compositions, liquid solutions, wound coverage systems, fibrous scaffolds, film-based materials, and implantable constructs [25,26,27]. Fibroin in various forms has demonstrated promising proliferative and regenerative properties in both cell culture experiments [13,17,28,29] and in vivo animal studies [13,18,30,31,32]. Accordingly, silk fibroin-based ointments, gels, and suspensions are regarded as highly promising for medical applications. Among these, gel formulations have attracted particular interest due to their ability to preserve the structural integrity of protein microparticles over extended storage periods, making them suitable candidates for regenerative therapeutic systems. Numerous studies have demonstrated the excellent biocompatibility and pronounced regenerative potential of silk fibroin microparticles, which provide a favorable microenvironment conducive to cellular adhesion and proliferation [30]. Owing to their physicochemical stability and combination of beneficial biological properties, such protein microparticles represent a compelling platform for the development of localized regenerative treatments.

There are numerous methods for producing silk fibroin microparticles. These include mechanical grinding [33], micelle formation in aqueous solutions containing polyethylene oxide [7], and protein precipitation in alcohol [6,34]. Each of these approaches enables the formation of particles with varying sizes and structural characteristics, which is of particular importance for their subsequent biomedical applications. Among these techniques, fibroin precipitation in alcoholic solutions is considered one of the most effective methods for obtaining stable protein particles with well-defined physicochemical properties.

The aim of the present study was to develop a carboxymethyl cellulose-based gel incorporating silk fibroin microparticles and to investigate its key properties, including the kinematic viscosity, fibroin particle size, cytotoxicity, and wound-healing activity of the resulting suspension. The obtained results may serve as a foundation for the development of new biomaterials in the fields of tissue engineering and regenerative medicine.

## 2. Materials and Methods

### 2.1. Preparation of Silk Fibroin Solution

Silk fibroin solution was obtained from *Bombyx mori* silkworm cocoons according to previously described methods with slight modifications [31,35]. The cocoons were shredded and washed in a 2.5 mg/mL sodium bicarbonate solution in distilled water (total volume 50 mL) at 80 °C for 80 min. After degumming, the material was rinsed to remove residual salts by three cycles of washing in distilled water at 99 °C for 30 min, followed by rinsing in 300 mL of distilled water at 20 °C between cycles. The cleaned material was then thoroughly dried at 85 °C. The dissolution of purified silk fibroin was carried out using a mixture of distilled water, 95% ethanol, and calcium chloride (Sharlab S.L., Barcelona, Spain) at a molar ratio of 8:2:1, respectively. The solution was prepared using 1 mL of the mixture per 200 mg of fibroin. Dissolution was performed in a sealed tube under continuous stirring at 90 °C for 40 min until a homogeneous solution was obtained. The resulting solution was purified from calcium chloride and ethanol by tenfold dialysis in distilled water at 20 °C, with 30 min intervals between water changes. The dialyzed fibroin solution was further clarified by centrifugation at 3000 rpm for 15 min using a SIGMA 6K10 2000W centrifuge (Sigma Laborzentrifugen GmbH, Osterode am Harz, Germany) to remove insoluble particles.

### 2.2. Preparation of Silk Fibroin Microparticles

We pre-cooled 95% ethanol to −20 °C. The fibroin solution was added dropwise (approximately 20 µL per drop) into the chilled ethanol to ensure uniform and complete protein denaturation. The resulting precipitate was separated by centrifugation at 3000 rpm for 15 min. The supernatant was removed and replaced with phosphate-buffered saline (PBS), and the protein pellet was mechanically resuspended. This washing procedure was repeated once more to further remove residual ethanol. The final protein precipitate was collected and reserved for further use.

### 2.3. Preparation of Composite Gel

The gel was prepared by mixing fibroin microparticles with a sodium carboxymethyl cellulose solution (Pharmaffiliates Private Limited, Panchkula, India) in phosphate-buffered saline. The CMC was initially dissolved in PBS at a concentration of 12 mg/mL under continuous stirring and controlled heating (up to 50 °C) to ensure complete dissolution. Subsequently, the fibroin suspension was combined with the CMC solution at a 1:1 (*v*/*v*) ratio, yielding a final composite gel containing 20 mg/mL fibroin and 6 mg/mL CMC.

The gel was sterilized by autoclaving at 121 °C for 15 min using a Sanyo MLS-3020U autoclave (Sanyo Electric Co., Osaka, Japan).

### 2.4. Kinematic Viscosity Measurement

The kinematic viscosity of the gel was measured using a U-tube viscometer (capillary diameter was 2 mm) according to established protocols [36]. The gel was introduced into the capillary of the viscometer, and the flow time of the liquid was recorded. The kinematic viscosity was calculated using the standard formula based on the recorded flow time and the viscometer constant, as shown in Equation (1):

(1) $$\begin{array}{c} \nu =\frac{g}{9.807\frac{m}{s^{2}}}\;\cdot K\;\cdot T, \end{array}$$

where ν is the kinematic viscosity (mm^2^/s), K is the viscometer constant (mm^2^/s^2^), T is the average flow time (s), and g is the acceleration due to gravity (m/s^2^).

Prior to measurement, the viscometer was calibrated using a fluid with known viscosity.

### 2.5. Gel Morphology Analysis

The physical appearance and morphological characteristics of the composite gel were assessed by visual inspection and photographic documentation. The gel was evaluated for homogeneity, consistency, and color immediately after preparation and then at monthly intervals throughout a 12-month storage period at 10 °C.

### 2.6. Preparations of Fibroin Microparticles for Scanning Electron Microscopy (SEM) Analysis

Samples of fibroin microparticles were dehydrated by transfer through ethanol solutions with increasing concentrations of 10, 20, 50, 70, and 96% for 30 min at each concentration. After dehydration, the suspension of fibroin microparticles was deposited on cover glass and dried in vacuum conditions for 1 h with use of the chamber of a Q150R ES rotary-pumped coating system (Quorum Technologies, Lewes, UK). The dried samples of fibroin microparticles on the glass were coated with a gold layer with a thickness of 5 nm in an argon atmosphere at an ion current of 20 mA and pressure of 1 mbar by the Q150R ES rotary-pumped coating system (Quorum Technologies, Lewes, UK).

### 2.7. Analysis of Fibroin Microparticles by SEM

The samples of fibroin microparticles on glass substrates coated with a 5 nm gold layer were analyzed using the Tescan Vega3 SBU scanning electron microscope (Tescan, Brno, Czech Republic). SEM measurements were performed in vacuum conditions on a microscope column in BSE (backscattering electron) mode with an operating voltage of 30 kV, working distance of 10.9 mm, and effective magnification of 4 × 10^3^. Image acquisition was performed with use of VegaTC software version 4.2.17.0 (Tescan, Brno, Czech Republic).

### 2.8. Cytotoxicity Assessment of the Suspension and Its Components

The cytotoxicity assessment of the suspension and its components was performed using the 3T3 cell line. Cells were seeded in 96-well plates at a density of 6 × 10^3^ cells per well and incubated at 37 °C for 24 h. The cell culture medium consisted of 10% fetal bovine serum (Gibco, Waltham, MA, USA), 0.1% gentamicin (HiMedia Laboratories LLC, Kennett Square, PA, USA), 1% Glutamax (Gibco, Waltham, MA, USA), and 89% Iscove’s Modified Dulbecco’s Medium (IMDM, Gibco, Waltham, MA, USA). For the assay, the suspension was mixed with culture medium at different volume ratios (25%, 12%, and 6%) and added to the wells. Subsequently, 50 μL of MTT solution (5×) was introduced into each well and incubated at 37 °C for 3 h. Following incubation, the MTT solution was removed, and 100 μL of DMSO was added to dissolve the formazan crystals. Absorbance was measured at 570 nm using a Feyond-A400 spectrophotometer (Hangzhou Allsheng Instruments Co., Ltd., Hangzhou, China). Cytotoxicity was determined based on cell viability relative to that of the control group.

### 2.9. Assessment of the Gel’s Functional Activity

The functional activity of the gel was evaluated using a full-thickness skin wound model in Wistar rats. All experiments with animals were approved by the Local Ethical Committee of the V.I. Shumakov National Medical Research Center of Transplantology and Artificial Organs. The experiments were conducted in accordance with Directive 2010/63/EU of the European Convention for the Protection of Vertebrate Animals Used for Experimental and other Scientific Purposes. The animals were housed individually under standard conditions: a temperature of 22 ± 2 °C, a humidity of 50–60%, and a 12-h light/dark cycle, with ad libitum access to food and water. A full-thickness skin defect measuring 12 mm^2^ was created on the dorsal surface of each animal, including removal of the epidermis, dermis, and part of the subcutaneous tissue. In the experimental group, the wound surface was treated with the gel prepared as described above. The gel was evenly distributed using a sterile spatula, and the wound area was covered with a sterile Tegaderm Film dressing (3M Co., St. Paul, MN, USA) to maintain a sealed environment and prevent external contamination. The gel was reapplied daily throughout the experimental period. Wound healing dynamics were assessed by regularly measuring the wound area. Healing efficiency was evaluated based on the percentage reduction in wound surface area relative to the initial measurement. The area reduction was calculated according to Equation (2):

(2) $$\begin{array}{c} R=\frac{S_{0}-S_{t}}{S_{0}}\times 100, \end{array}$$

where *R* is the wound healing percentage (%), *S*_0_ is the initial wound area (mm^2^), and *S_t_* is the wound area on the observation day (mm^2^).

### 2.10. Histological Evaluation of Tissue Regeneration

After explantation, the regenerated tissue samples were fixed in 10% neutral buffered formalin for a minimum of 24 h at room temperature. Standard histological processing was subsequently performed, including dehydration through a graded ethanol series (50%, 60%, 70%, 80%, and 96%), followed by treatment with a mixture of ethanol and chloroform and two changes of absolute chloroform. The samples were then embedded in paraffin. Serial sections 5–6 μm in thickness were obtained using a rotary microtome RM2245 (Leica Microsystems GmbH, Wetzlar, Germany). To assess tissue morphology, the sections were stained with Mayer’s hematoxylin and eosin (BioVitrum, Saint-Petersburg, Russia), and Masson’s trichrome stain (BioVitrum, Saint-Petersburg, Russia) was used for visualization of collagen fibers. Microscopic analysis was performed using an Eclipse 50i light microscope (Nikon, Tokyo, Japan) equipped with a digital camera. The assessment focused on the degree of epidermal and dermal regeneration and the overall restoration of skin architecture.

### 2.11. Data Analysis

All quantitative data are presented as the mean ± standard deviation (M ± SD). Statistical significance between experimental groups was evaluated using the non-parametric Mann–Whitney U test. Differences were considered statistically significant at *p* < 0.05. Data processing and graphical representation were performed using OriginPro software version 2024b (OriginLab Corporation, Northampton, MA, USA).

## 3. Results

### 3.1. Preparation of Fibroin Particles

In the present study, an aqueous colloidal solution containing silk fibroin microparticles ranging from 1 to 20 µm was successfully developed. A major advantage of the resulting formulation was its exceptional physicochemical stability. When stored at 10 °C, the system remained stable for over one year without visible sedimentation, discoloration, changes in viscosity, or spontaneous gelation. This level of stability notably exceeds that of conventional silk fibroin solutions, which typically exhibit gelation, aggregation, and loss of homogeneity within 2–4 weeks under similar storage conditions [32].

**Figure 1:**
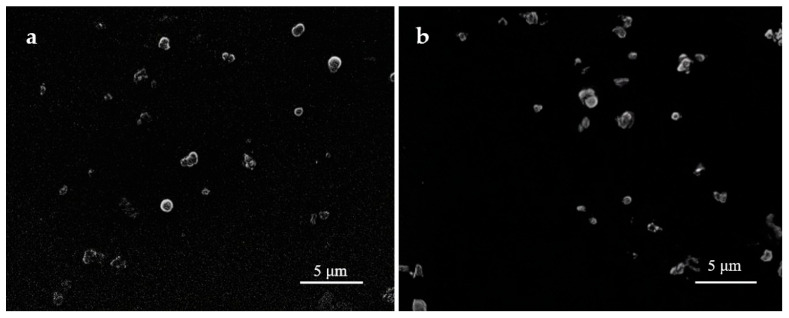
SEM images of silk fibroin microparticles. (**a**) Microparticles obtained one day after preparation. (**b**) Microparticles after one year of storage at 10 °C.

**Figure 2:**
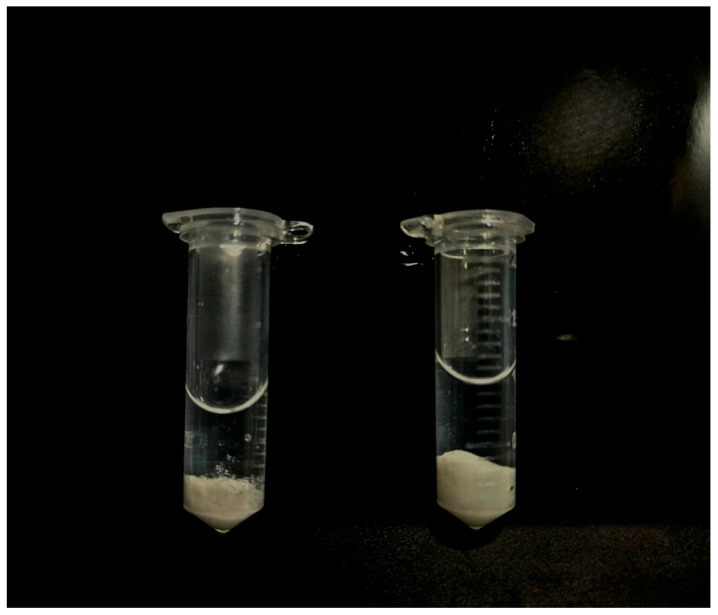
Macroscopic photographs of the silk fibroin microparticle dispersion in PBS: freshly prepared sample (**left**) and sample after 12 months of storage at 10 °C in PBS (**right**).

Figure 1 presents SEM images of the silk fibroin microparticles obtained one day after preparation (Figure 1a) and after 12 months of storage at 10 °C (Figure 1b), demonstrating that the size and morphology of the particles remained unchanged. In addition, macroscopic images of the colloidal dispersion (Figure 2) visually confirm the absence of color shift, turbidity, or self-gelation.

The high stability of the obtained fibroin solution enables the development of durable systems with precisely tunable viscous properties. Viscosity can be accurately adjusted by adding thickeners without the risk of unpredictable changes in consistency over time.

### 3.2. Gel Morphology

The gel was formulated by incorporating carboxymethyl cellulose (CMC) as a thickening agent. The selection of CMC was based on its high hydrophilicity, moisture retention capacity, and ability to form stable gel-like structures upon mixing with water [37]. These properties allow for the maintenance of an optimal moist environment at the wound site, thereby promoting tissue regeneration [38]. Additionally, CMC exhibits excellent biocompatibility, physiological inertness, and non-toxicity, making it a safe material for biomedical applications [39]. Previous studies have demonstrated that CMC-based hydrogels support cell viability, exhibit favorable viscosity and mechanical characteristics, and enhance wound healing, particularly when supplemented with bioactive compounds [39,40]. These features determined the choice of CMC as the matrix for gel preparation in the present study, where it acted as a carrier for silk fibroin microparticles, forming a convenient, stable, and biologically neutral platform for topical application. The gel formation mechanism is primarily based on physical interactions, including the entanglement of CMC polymer chains and hydrogen bonding both within the CMC matrix and between CMC and silk fibroin microparticles. These interactions result in a viscous, physically stable system without chemical crosslinking, which maintains fluidity suitable for easy application while preventing rapid spreading.

The resulting gel is a viscous, homogeneous white mass with uniformly dispersed fibroin particles. It possesses a soft, pliable consistency similar to ointments. Unlike rigid hydrogels, this formulation conforms well to the contours of the application site. The measured kinematic viscosity of the gel was 36.5 × 10^−6^ St. This level of viscosity plays a crucial role in the technological performance of the material, allowing it to maintain plasticity and enabling uniform application either by surface spreading or via syringe. The high viscosity also prevents spontaneous spreading.

**Figure 3:**
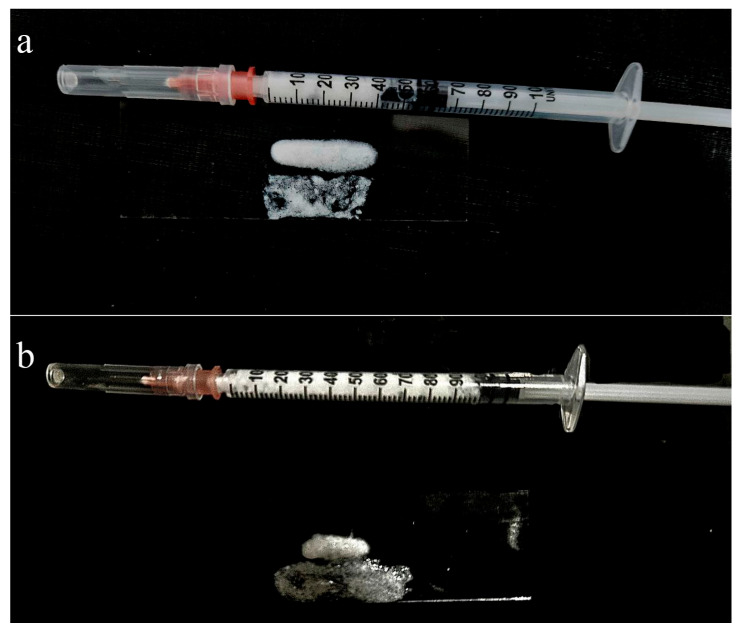
Visual presentation of the composite gel being dispensed from a syringe and spread with a spatula: a freshly prepared sample (**a**) and the sample after 12 months of storage at 10 °C (**b**).

Figure 3 illustrates three methods of gel application: injection through a fine-gauge needle, extrusion from a syringe, and manual spreading with a spatula. In all cases, the gel retains its structure without dripping or uncontrolled flow, demonstrating excellent cohesiveness and ease of handling.

In addition to characterizing the stability of the colloidal silk fibroin microparticle suspension, the long-term integrity of the complete composite gel was also systematically evaluated. Over the course of 12 months, samples of the gel stored at 10 °C were periodically examined for visual and physicochemical changes. No signs of sedimentation, phase separation, discoloration, or microbial contamination were observed. Kinematic viscosity was measured at three-month intervals and remained consistent throughout the observation period.

The optimized viscous properties of the gel may enhance its interaction with biological tissues by ensuring mechanical stability at the application site and creating a favorable environment for the transport of nutrients and bioactive molecules. Thus, the developed gel exhibits promising features for potential use in medicine and bioengineering.

### 3.3. Cytotoxicity and Biocompatibility Assessment of the Gel

**Figure 4:**
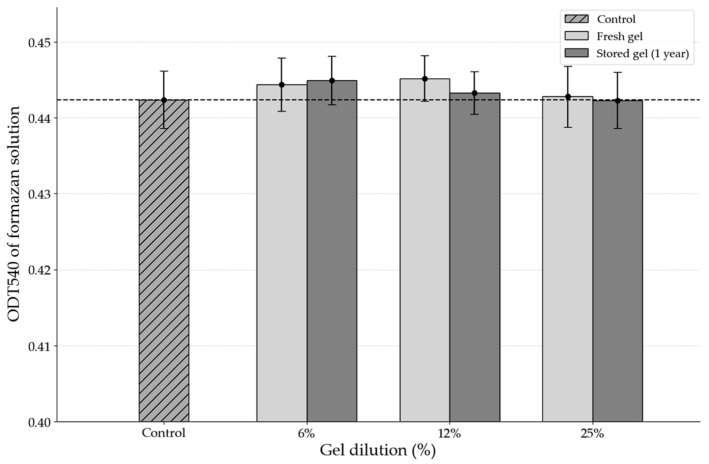
Comparative cytotoxicity of the gel and control group in 3T3 cells.

According to the results of the MTT assay, the gel did not exert any negative effects on cell viability or metabolic activity. The optical density of the formazan product, which was proportional to the number of viable cells, showed no statistically significant difference between the experimental groups treated with various dilutions of the gel and the control group undergoing natural regeneration without intervention (Figure 4). This indicates that cellular metabolism was preserved at levels comparable to those of the control, with no detectable toxic effects from the gel components.

Importantly, even after prolonged cell–gel contact, no significant reduction in cell viability was observed, suggesting the absence of any cumulative cytotoxic effects.

Microscopic examination further revealed no pathological changes in cell morphology in the presence of the gel. The cells retained their characteristic fibroblast-like shape typical of the 3T3 line, displayed active proliferation, and exhibited no signs of apoptosis or necrosis. These findings confirm the biocompatibility of the material and highlight its potential for applications in tissue engineering, regenerative medicine, and localized delivery of bioactive agents.

To evaluate long-term biocompatibility, the same tests were repeated using gel samples that had been stored for 12 months under standard conditions (10 °C). The results demonstrated that the aged gel retained full biological activity and cytocompatibility: there were no measurable differences in cell viability, morphology, or proliferation rate compared with the freshly prepared gel. These findings confirm not only the absence of toxic degradation products over time but also the remarkable preservation of biological functionality, further supporting the hydrogel’s suitability for clinical and biotechnological applications.

### 3.4. Functional Activity of the Gel

**Figure 5:**
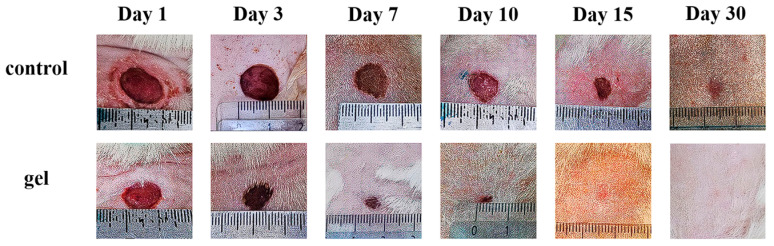
Macroscopic images of wounds in the control and experimental groups captured at various stages of healing.

The application of the gel containing silk fibroin microparticles in an experimental model of full-thickness skin defect demonstrated a pronounced acceleration of tissue regeneration. Complete re-epithelialization in the experimental group was observed by day 15, whereas in the control group, wound closure occurred only by day 30. The reduction in the wound area progressed significantly faster with gel treatment, as confirmed by regular measurements (Figure 5) and calculations of wound closure percentage (Table 1). An additional control group treated with carboxymethylcellulose (CMC) gel without fibroin showed delayed healing, with full wound closure achieved by day 23. The reduction in wound area progressed significantly faster with fibroin-containing gel treatment, as confirmed by regular measurements.

**Figure 6:**
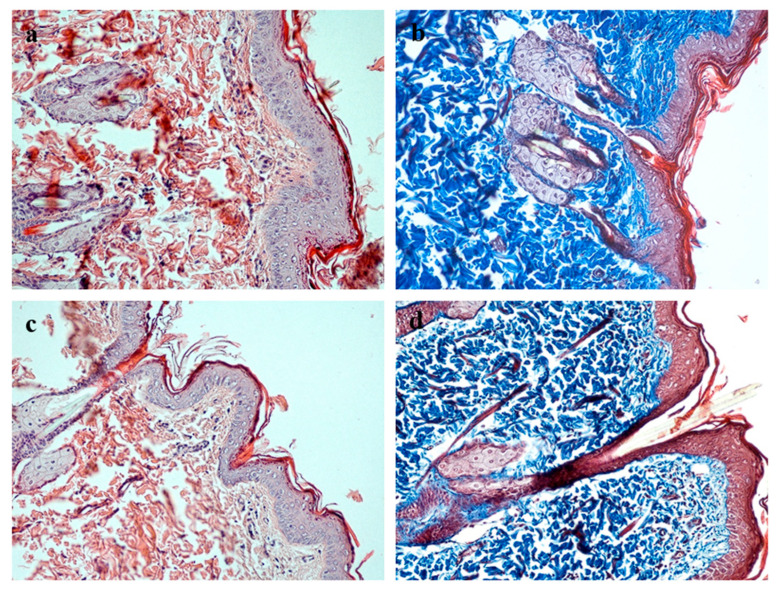
Comparative histological analysis of the healed skin area in the experimental group and intact rat skin. Magnification ×200. (**a**,**b**) Healed skin defect on day 15 in the experimental group: stained with hematoxylin and eosin (**a**) and Masson’s trichrome (**b**); (**c**,**d**) intact skin stained with hematoxylin and eosin (**c**) and Masson’s trichrome (**d**).

Histological examination of the healed full-thickness skin defects in the experimental group revealed the complete restoration of all morphological skin layers (Figure 6). A well-organized, multilayered epithelium formed over the wound site, with clearly delineated basal, spinous, granular, and cornified layers comparable in thickness and architecture to those of intact skin. The underlying connective tissue comprised mature, fibrous dermis containing sebaceous glands and hair follicles. Collagen fibers stained by Masson’s trichrome displayed dense packing and orientation characteristic of normal dermis. No signs of chronic inflammation or fibrotic remodeling were detected in the regenerating tissue. The newly formed skin integrated seamlessly with the surrounding intact tissue, with no distinct boundary between the repair zone and adjacent skin.

**Figure 7:**
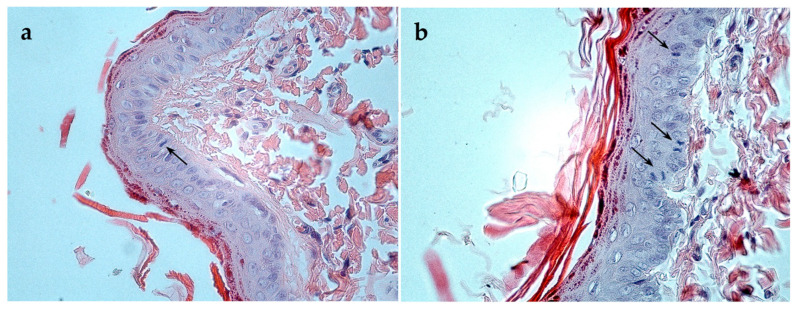
Proliferative activity of the basal layer in regenerated epidermis. Mitotically dividing cells are indicated with arrows. Hematoxylin and eosin staining, magnification ×400. (**a**) Intact skin, (**b**) gel treatment.

Moderate mitotic activity was observed in the basal layer (Figure 7), indicating ongoing keratinocyte proliferation and the maintenance of epithelial homeostasis; notably, the number of mitotic figures in the experimental group exceeded that in the control group.

Thus, the morphological findings indicate the complete regeneration of the skin process, with the restoration of normal tissue architecture closely resembling that of intact skin, thereby confirming the efficacy of the proposed biopolymer system.

## 4. Discussion

The results of the present study demonstrate that the developed carboxymethyl cellulose-based gel with fibroin microparticles possesses a combination of physicochemical and biological properties that make it a promising prospect for use in regenerative medicine. The hydrogel exhibited a homogeneous distribution of silk fibroin microparticles (1–20 µm) within the gel, which is essential for maintaining uniform functional activity and ensuring predictable behavior during application.

One of the key advantages of the proposed system is its exceptional long-term stability. Unlike conventional fibroin solutions, which tend to undergo spontaneous gelation or aggregation within 2–4 weeks [32], the gel prepared using the current methodology retained its homogeneity and functional properties for over one year when stored at 10 °C. This improvement in shelf-life has considerable practical implications, facilitating transportation, storage, and clinical deployment without compromising performance.

The measured kinematic viscosity (36.5 × 10^−6^ St) indicates that the hydrogel is sufficiently fluid for topical or invasive application, while maintaining structural integrity and avoiding unintended spreading.

In vitro cytotoxicity assays using 3T3 fibroblasts confirmed the biocompatibility of the hydrogel, showing no significant reduction in cell viability compared to untreated controls. This aligns with previous reports highlighting the low immunogenicity and high cytocompatibility of silk fibroin materials [1,2,3,4] and supports its potential as a safe system for tissue regeneration.

The in vivo experiments further validated the regenerative potential of the developed hydrogel. In a full-thickness skin wound model in rats, the application of the fibroin-based hydrogel resulted in complete re-epithelialization by day 15, which was substantially faster than the 30-day healing period observed in the untreated control group. Furthermore, a separate control group treated with carboxymethylcellulose (CMC)-only hydrogel—representing the base formulation without active fibroin—showed wound closure by day 23. This difference clearly highlights the therapeutic contribution of silk fibroin microparticles to the accelerated healing process.

Histological examination confirmed the restoration of all skin layers, with the tissue architecture approaching that of intact skin. Notably, moderate mitotic activity was detected in the basal layer of the regenerated epidermis, particularly in the experimental group, indicating active keratinocyte proliferation. In contrast, mitotic figures were scarce in intact (non-injured) skin, suggesting that the observed cell division was indeed part of the regenerative response induced by the hydrogel. These findings further support the notion that silk fibroin microparticles create a microenvironment conducive to cell proliferation and tissue regeneration [6,14,17].

Despite the promising results obtained in this study, several limitations should be acknowledged. The experiments were limited to a single animal model and short-term observation period. Additional studies are required to assess long-term outcomes, immune responses, and performance in more clinically relevant models. Future work should also focus on optimizing the formulation and exploring the incorporation of therapeutic agents to further enhance the gel’s regenerative potential.

Overall, the developed hydrogel not only meets the physicochemical and biological criteria for a wound healing scaffold but also demonstrates practical advantages in terms of storage stability and ease of use. These properties, along with the demonstrated efficacy in both in vitro and in vivo systems, position this material as a strong candidate for clinical translation in the fields of wound management, skin repair, and tissue engineering.

## 5. Conclusions

A silk fibroin-based hydrogel with favorable physicochemical and biological properties was successfully developed in this study. The material demonstrated long-term stability, suitable characteristics for topical application, and excellent biocompatibility. In vivo experiments confirmed its ability to significantly accelerate skin regeneration in a full-thickness wound model. These results suggest that the proposed hydrogel holds strong potential for use in regenerative medicine and wound healing applications.

## Figures and Tables

**Table 1:** Comparative dynamics of wound area reduction in the control and experimental groups.

	Percentage Reduction in Wound Area (%)				
	Day 1	Day 3	Day 7	Day 10	Day 15
control	0	0	16.4 ± 2.3	43.9 ± 6.1	75.1 ± 3.8
CMC	0	7.5 ± 6.1	24.7 ± 4.6	51.2 ± 5.3	88.9 ± 4.3
gel	0	30.8 ± 5.4	74.2 ± 4.7	89.1 ± 3.4	100

## Data Availability

The original contributions presented in this study are included in the article. Further inquiries can be directed to the corresponding authors.

## Abbreviations

The following abbreviations are used in this manuscript:

CMC	Carboxymethyl cellulose
SEM	Scanning Electron Microscopy
PBS	Phosphate-buffered saline
